# Numerical and Experimental Analyses of a Phase Change Material-Thermoelectric System Integrated with a Heat Sink and Radiative Cooling

**DOI:** 10.1021/acsami.4c17331

**Published:** 2024-12-13

**Authors:** Aminu Yusuf, Sedat Ballikaya

**Affiliations:** † Department of Engineering Sciences, Istanbul University−Cerrahpasa, Avcilar, Istanbul 34320, Turkey

**Keywords:** energy harvesting, long-term operation, phase change material, radiative cooling, sustainable power system, thermoelectric generator

## Abstract

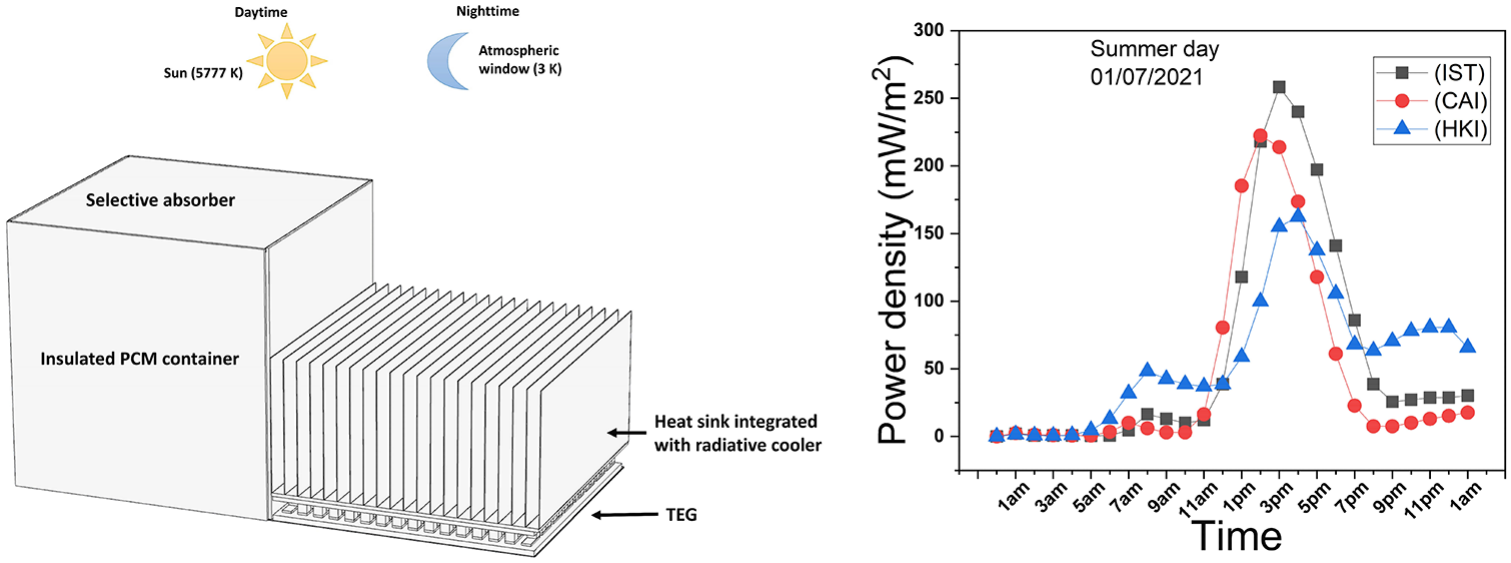

Providing power to remotely located sensors can pose significant challenges, especially when these sensors are positioned in the open sea or remote wilderness. The development of a durable, low-maintenance power system with an extended lifespan is of utmost importance, and this study is primarily motivated by this need. This research focuses on the design, modeling, and development of a system that combines a phase change material-thermoelectric generator (PCM-TEG) with a heat sink-coated radiative cooler (HS-RC). This integration yields a remarkable 10-fold increase in performance compared to relying solely on radiative cooling. Additionally, the study highlights the substantial influence of the PCM’s melting temperature on the TEG’s nighttime operation. To ensure the TEG operates effectively during nighttime hours, it is imperative that the PCM can release energy. Furthermore, the study emphasizes the importance of a radiative cooler with high reflectivity across the entire solar spectrum to achieve robust diurnal radiative cooling performance. On a summer day, the PCM-TE-RC-HS system showcases its ability to generate substantial power densities, reaching maximum values of 258 mW/m^2^ in Istanbul, 222 mW/m^2^ in Cairo, and 162 mW/m^2^ in Helsinki.

## 1 Introduction

The importance of powering low-energy sensors has grown significantly, given the exponential increase in the number of remote sensors.^1^ These sensors serve a variety of functions, including measuring weather parameters, detecting intrusions, tracking Internet usage, and determining locations, among others. Due to their low energy consumption and their widespread distribution in remote locations, connecting power cables to these sensors is not economically feasible. While batteries can be used to supply power to the sensors, the frequent need for recharging, discharging, and battery replacement fails to provide a comprehensive solution.^2^ Given that, there is a continuous surge of interest in low-grade power energy harvesters, driven by their ability to power various sensors and low-energy devices. Among these energy harvesters, the thermoelectric generator (TEG) stands out as it can convert thermal energy into electrical energy.^3^ TEGs are environmentally friendly devices that operate silently day and night without emitting pollution.^4^ A self-contained remote sensor system can be designed to incorporate TEGs alongside a battery bank and sensors. In this setup, the power generated by the TEGs can be harnessed to recharge the batteries, which, in turn, supply power to the sensors. Despite the advantages of TEGs, their relatively low energy conversion efficiency makes them less appealing, and, as a result, this aspect is a key area of interest for researchers.^5^

The functionality of a TEG is influenced by various factors, including the properties of thermoelectric (TE) materials, contact resistances, heat transfer losses, and load resistance, as well as the TEG’s geometry.^6^ It is desired that the TE materials should possess high values of electrical conductivity and Seebeck coefficient simultaneously, along with low thermal conductivity. This combination results in a high figure of merit, a parameter used to evaluate TE material performance.^7^ Conversely, efforts should be made to minimize electrical and thermal contact resistances, as well as radiation and convection losses.^8^ During the development stage of the module, pressure can be applied to reduce the contact resistivity induced by thermal expansion.^9^ Ensuring the optimal geometry of a TEG is just as crucial as identifying a suitable material with high TE performance. This is because optimizing the TEG’s geometry not only enhances its thermoelectric efficiency but also improves its mechanical stability.^10^ For instance, when comparing two micro-TEGs with different leg geometries (filled and hollow), the micro-TEG featuring hollow leg geometry outperforms the one with filled geometry, for the same TE leg length and cross-sectional area.^11^ This is because the leg geometry significantly influences the temperature distribution within the device. Maduabuchi et al.^12^ conducted an optimization of a TEG’s geometry, resulting in a 78% increase in its maximum efficiency compared to before optimization. Moreover, the optimization led to a 73% reduction in thermal stress, indicating improvements in mechanical reliability and lifespan. Zhu et al.^13^ coupled genetic algorithm with an artificial neural network to optimize the geometry of a segmented thermoelectric generator. This optimization proved to be as accurate as the finite element method and significantly faster than the latter. The integration of machine learning in the TEG design and optimization represents a noteworthy advancement in the field of energy harvesting.

In the context of a 24-h remote application, ensuring a continuous energy supply on the hot side of the TEG is crucial. However, conventional solar TEGs face challenges in maintaining consistent energy output due to fluctuations in solar radiation throughout the day and the absence of solar radiation at night. To address this issue, one effective solution involves integrating the hot side of the TEG with a phase change material (PCM). A PCM is a substance capable of absorbing or releasing latent energy during phase transitions, typically occurring at a constant temperature.^14^ Both the PCM and the TEG experience thermal cycling during operation, necessitating the need for chemical and mechanical stability in the combined system. An ideal PCM should exhibit qualities such as a high crystallization rate, excellent thermal conductivity, substantial latent heat capacity, cost-effectiveness, abundance, and long-term reusability. A PCM on the hot side of a TEG can protect the TEG from excessive exposure to high temperatures, which might damage the TEG and ensure a steady and continuous supply of thermal energy for the TEG’s operation.^15^ On the other hand, PCM absorbs and stores thermal energy from the cold side when placed on the cold side. A typical example of this integration can be found in a building application, where, during the daytime, the hot side of the TEG absorbs solar radiation while the PCM absorbs the thermal energy to provide cooling on the cold side.^16^ At night, the energy stored by the PCM during the day is released to the cold side of the TEG. A similar arrangement has been demonstrated in systems that incorporate a photovoltaic (PV) cell, sandwiching the TEG between the PV and the PCM.^17^ Furthermore, to enhance TEG performance, microchannel heat pipes have been introduced into the PCM container, improving heat transfer, and allowing for more efficient absorption of heat from the TEG’s cold side.^18^

The temperature of the Sun (5777 K) and that of outer space (3 K) are the reservoirs of heat and cold, respectively.^19,20^ Utilizing the cold of outer space for cooling the cold side of a TEG is made possible by emitting thermal energy into the universe through a wavelength in the range of 8 μm - 13 μm (sky window). This is known as radiative sky cooling, and it differs from conventional cooling methods, as it releases excess heat into outer space without any energy consumption.^21^ A radiative cooler (RC) can be designed to provide cooling both during the day and night.^22^ The RC reflects incoming solar energy during the day, while at night, it emits thermal energy into the frigid expanse of outer space via the sky window.^23^ To ensure the continuous operation of a TEG, it can be integrated with a selective absorber and radiative cooler on its hot and cold sides, respectively.^24^ Indoor experiments of this study revealed nighttime and daytime power densities of 4 mWm^–2^ and 489 mWm^–2^, respectively. Similarly, the outdoor experiments produced nighttime and daytime power densities of 0.8 mWm^–2^ and 91 mWm^–2^, respectively. For wearable TEGs, the proper design of a heat sink is critical for satisfactory performance. Comparing the performance of three TEGs: one with a bare cold side, one with a finned cold side, and one with a radiative cooling-coated cold side, the TEG with radiative cooling exhibits superior performance.^25^ The performance of a concentrated TEG-RC system is determined both theoretically and experimentally.^26^ The study revealed that concentrating solar energy and increasing the radiative cooler’s surface area enhanced the output power, while higher wind speed negatively affected the radiative cooling performance, resulting in the reduction of the output power from 8.58 mW to 6.96 mW. Radiative cooling alone may not be sufficient to cool the cold side of a TEG due to the substantial amount of energy that needs to be dissipated rapidly. Integrating a radiative cooler with a heat sink can enhance cooling performance by combining convection cooling with radiative cooling.^27^

While numerous studies have analyzed various TEG system configurations, such as PV–PCM-TE system,^28−30^ PCM-TE system,^31,32^ and TEGs integrated with radiative cooling,^33−35^ it is currently not clear in the literature how a PCM-TEG integrated with both a heat sink and radiative cooling technology behaves. This information is especially needed given the focus on operating the PCM-TEG system continuously day and night. To address this research gap, this study introduces a novel structure of a PCM-TEG-HS integrated with radiative cooling technology. For effective radiative cooling, the radiative cooling surface (a heat sink coated with radiative cooling paint) must face the sky, which is why the current configuration was chosen. It is also noted that exposure to sunlight adversely impacts the cooling performance of the heat sink with radiative cooling paint. However, a further optimization of the radiative cooling paint will improve its cooling performance. The PCM provides a continuous thermal energy supply to the TEG’s hot side, at the same time the cold side is kept cool by the heat sink-coated radiative cooler, thus, ensuring 24-h operation. The integration of the heat sink with radiative cooling serves as an optimization of the cooling performance because the system utilizes both convection and radiative cooling on the cold side. Furthermore, the study evaluates the performance of the PCM-TE-RC system with and without a heat sink, investigates the impact of the PCM’s melting temperature, and analyses the effect of the radiative cooler’s emissivity on system performance. Moreover, it explores the feasibility of 24-h operation in different cities with varying solar insolation in summer and winter.

## 2 Theoretical Background

**Figure 1:**
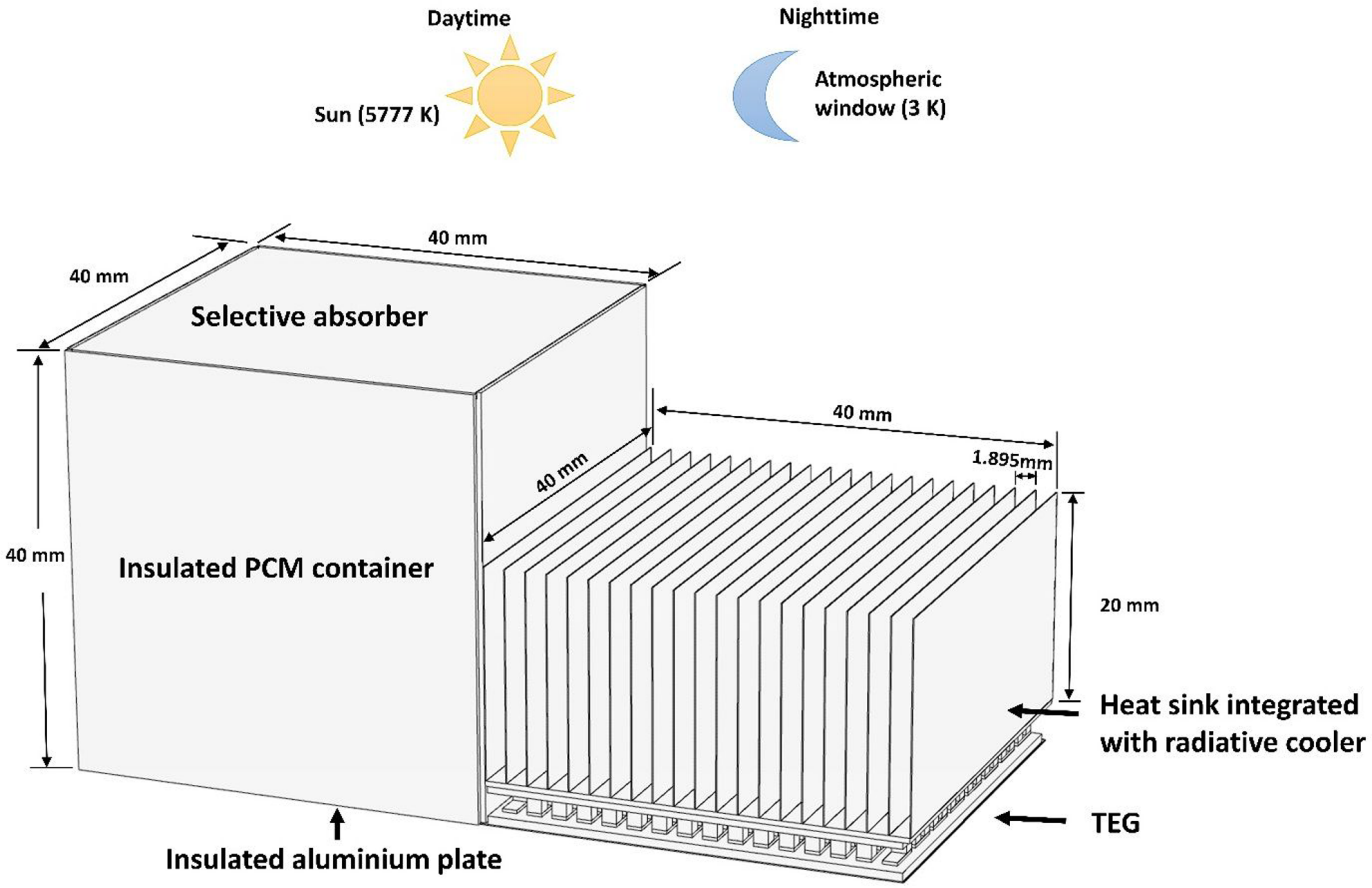
Proposed PCM-TE-HS-RC system.

The system comprises an insulated container for PCM, an aluminum plate, a TEG, and a heat sink coated with a radiative cooler, as depicted in Figure 1. The PCM receives and stores solar energy with the assistance of a selective absorber with 90% absorption efficiency. Simultaneously, the TEG absorbs thermal energy from the PCM, a process facilitated by an aluminum plate attached to the base of the PCM container. The TEG can generate power, and this generation can be further optimized by integrating the cold side with either a heat sink or a radiative cooler. An additional enhancement in power generation is achieved by coating the heat sink with the radiative cooler. This approach leverages convection and radiative cooling to enhance the system’s performance.

### 2.1 Phase Change Material Model

In this study, the PCM is modeled with the apparent heat capacity method, treating it like a single-phase fluid with properties that vary. These properties differ between its solid and liquid states. To characterize the fluid within the transitional mushy region, where both solid and liquid phases coexist, an interpolation technique is applied. The PCM is modeled using the heat energy equation with a convective term given as
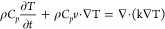
1where *k* is the thermal conductivity, *v* is the velocity of the fluid (zero when PCM is in solid form), ρ is the density, *T* is the temperature, and *C*_*p*_ is the heat capacity.

In the mushy region, the heat capacity is modeled as the apparent heat capacity given as^36^
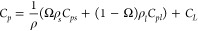
2where Ω is the liquid fraction, it is 0 when the PCM is in liquid form, 1 when the PCM is in solid form, and 0 < Ω < 1 during the phase transformation, *s* refers to the solid state, and *l* refers to the liquid phase. The last term in eq 2 is the latent heat distribution, which manages the heat absorption/dissipation during the phase transformation and is given as
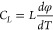
3where *L* and φ refer to the latent heat and the mass fraction, respectively. The mass fraction is defined as
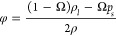
4

Eq 4 takes the values of −0.5 before the phase transformation and +0.5 after the phase transformation.

In the mushy region, other properties of the PCM such as the density (*ρ*_*eff*_) and thermal conductivity (*k*_*eff*_) are modeled as follows:
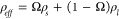
5
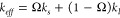
6

The phase transformation takes place over the range of *T*_*pcm*_*± ΔT/2*. Here, *T*_*pcm*_ is the PCM’s melting temperature and *ΔT* is the melting range. Table 1 gives the PCM’s properties.

**Table 1:** Properties of the RT28HC PCM^37^

Property	
ρ (kg m^3^)	770 (liquid), 880 (solid)
*k* (W m^–1^ K^–1^)	0.2
*L* (kJ kg^–1^)	250
*T*_*pcm*_ (K)	301
Δ*T* (K)	300–302
*C*_p_ (J kg^–1^ K^–1^)	2000
PCM container (mm^3^)	64,000

### 2.2 Thermoelectric Generator Model

The TEG works based on the Seebeck effect when a temperature gradient exists between its hot and cold surfaces electrical potential is generated. Several phenomena are included in the operation of a TEG and these include: Peltier heating and cooling, which significantly influence electrical generation; Joule heating and the Thomson effect, both of which counteract thermal energy absorption but contribute to its dissipation.^38^ Additionally, Fourier heat plays a role in facilitating heat transfer from the hot surface to the cold surface. Therefore, the thermoelectric phenomena can be defined by the conservation of energy and electric current continuity equations respectively given as
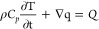
7
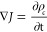
8

The heat flux in eq 7 contains both the Fourier heat and Peltier power:^39,40^
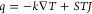
9

Likewise, the Joule heat in eq 7 is given as
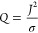
10

The current density is given as
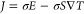
11

Substituting eqs 8–10 into eq 6, the thermoelectric-heat energy equation is derived as
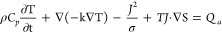
12where σ is the electrical conductivity, *S* is the Seebeck coefficient, *J* is the current density, *Q*_*a*_ is the internal heat source.

Eq 12 is used to model the heat transfer in thermometric generators. On the ceramic plates, the Seebeck and current density are zero; therefore, the joule heat (third) and Peltier power (fourth) terms are zero. For the case of electrical conductors, only the Peltier power is zero.

The electrical current flowing in the TEG can be computed as
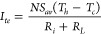
13where *R*_*L*_ is the load resistance, *N* is the number of TE couples, *S*_*av*_ is the average Seebeck coefficient, *R*_*i*_ is the total internal resistance of the module, *T*_*h*_ are *T*_*c*_ are the temperatures of the hot and cold sides.

Eqs 14 and 15 can be used to calculate the electrical power and power density of the TEG as
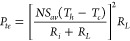
14
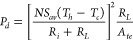
15where the total internal resistance is calculated from
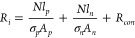
16where *R*_*con*_ is the contact resistance, *l* is the leg length, *A* is the area of a TE leg, and the subscripts *n* and *p* refer to the *n*- and *p*-type TE materials. Table 2 presents the TE material properties of the TEG.

**Table 2:** TE Properties of the TEG^40^

Property	Copper	Ceramic	Solder	n-leg	p-leg
ρ (kg m^–3^)	8930	3970	7240	7830	6810
σ (S m^–1^)	5.9 × 10^7^	1 × 10^–12^	2 × 10^7^	1.33 × 10^5^	1.25 × 10^5^
*C*_*p*_ (J kg^–1^ K^–1^)	386	800	210	154.4	207.6
*S* (μV/K)	—	—	—	–203.06	204.98
*k* (W m^–1^ K^–1^)	385	25	55	2.01	1.86
*l* (m)	1 × 10^–4^	5 × 10^–4^	1 × 10^–5^	1.6 × 10^–3^	1.6 × 10^–3^
*A* (m^2^)	5.32 × 10^–6^	1.6 × 10^–3^	1.96 × 10^–6^	1.96 × 10^–6^	1.96 × 10^–6^

### 2.3 Radiative Cooling Coated Heat Sink Model

The purpose of coating the heat sink (HS) with a radiative cooler (RC) is to enhance the cooling performance during the operation of the TEG. The RC reflects a significant portion of solar energy during daylight hours and emits thermal energy to the outer cold space at night. The energy balance for the RC in a steady state can be expressed as
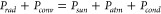
17where *P*_*rad*_ represents radiative power density, *P*_*conv*_ is the convective cooling power density associated with the heat sink, and *P*_*sun*_ is the fraction of solar power absorbed by the RC. Likewise, *P*_*atm*_ is the atmospheric power and *P*_*cond*_ is the heat transfer by conduction. Considering the radiative energy terms only, the net radiative cooling power can be expressed as
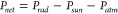
18

It is desired that the magnitude of the radiative cooling power exceeds the sum of solar and atmospheric power absorption. The radiative cooling power is expressed as^41^
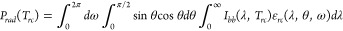
19where θ, ω, λ are the zenith angle, solid angle, and wavelength, *ε*_*rc*_ (*θ, λ*) is the RC’s emissivity, and the spectral radiance of a blackbody *I*_*bb*_ (*λ, T*_*rc*_) is given as^42^
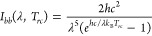
20where *h, k*_*B*_, and *c* are the Planck constant, Boltzmann constant, and the speed of light, respectively.

The solar energy and atmospheric power absorbed by the RC are respectively defined as
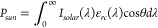
21
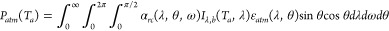
22where *I*_*λ,b*_ (*T*_*a*_*, λ*), *α*_*rc*_ (*λ, θ, ω*), and *ε*_*atm*_ (*λ,θ*) are the spectral irradiance of a blackbody, absorptivity of the RC, and the atmospheric emissivity, respectively. The atmospheric emissivity expressed by a piecewise function:^43,44^
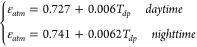
23where the dew point temperature (*T*_*dp*_) is in degrees Celsius. The zenith angle mentioned in eqs 19 and 22 indicate the position of the sun with the vertical axis directly above the observer; it can be determined from^45^
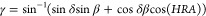
24
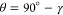
25where δ is the declination angle, β is the local latitude of the location, γ is the elevation, HRA is the hour angle. It can be seen that the zenith angle varies throughout the day and year, and it also depends on the local latitude of the location.

Since the RC is coated on a parallel plate heat sink, the convection and conduction terms in eq 17 strongly depend on the continuity, conservation of momentum, and conservation of energy equations, which modeled the heat sink and these equations are respectively given as^46^
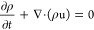
26
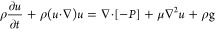
27
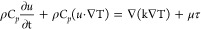
28where *P*, *u*, *g*, and μ are the static pressure, the dynamic viscosity of air, acceleration due to gravity, and the velocity vector, respectively, and τ is the viscosity loss.

The effectiveness of the heat sink can be determined by knowing the overall surface thermal efficiency, speed of air, and heat transfer coefficient. The thermal efficiency of the heat sink has an inverse correlation with its thermal resistance, that is the efficiency increases with a decrease in the thermal resistance. The heat sink’s thermal resistance is the summation of the thermal resistances of the base and fin array:
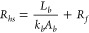
29

The effective thermal resistance of the fin array is then given as^47^
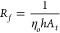
30where *η*_*0*_ is the thermal efficiency of the fin array, and *A*_*t*_ is the area of the fin and unfinned portion, these parameters are respectively given as
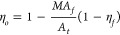
31
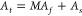
32where *h* is the heat transfer coefficient of air, *M* is the number of fins, *A*_*f*_ = *t* × *L*_*f*_ is the area of a fin, *A*_*s*_ = *A*_*b*_ – *MA*_*f*_ is the total area of the unfinned portion, *η*_*f*_ is the thermal efficiency of a fin, which is defined as
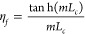
33where the corrected height of the fin *L*_*c*_ = *L* + (*t*/2), *L* is the height of the fin, *t* is the thickness of the fin, *L*_*f*_ is the length of the fin, and the constant , *k*_*b*_ is the thermal conductivity of the heat sink material, which is aluminum in this case.

Finally, the cooling provided by the heat sink can be computed as
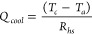
34

Now, t he total cooling on the cold side is the sum of the cooling from the radiative cooler (eq 18) and the parallel plate heat sink (eq 34), this proves that the cooling performance can be enhanced. The performance of the heat sink-coated radiative cooling is influenced by its design parameters, which directly affect heat dissipation, temperature gradient, and overall system efficiency. For example, the distance between the fins influences airflow and heat dissipation. Narrower spacing can increase the surface area for heat exchange but may restrict airflow, reducing convective heat transfer. Optimal spacing balances these factors to maximize cooling efficiency. Likewise, taller fins increase the surface area for heat transfer, enhancing heat dissipation. However, excessively tall fins may lead to structural instability. Similarly, fin thickness impacts thermal conduction and the heat sink’s overall weight and material usage. The base plate should be designed to effectively spread the heat from the TE module to the fins. A thicker base improves thermal spreading but adds thermal mass, which can delay system responsiveness. The heat sink material affects thermal conductivity. Common materials like aluminum and copper offer high thermal conductivity, with copper providing superior performance but higher cost and weight. The heat sink’s orientation and interaction with ambient airflow (natural or forced convection) impact its performance. Proper alignment ensures efficient heat removal from the hot side of the TE module. Lastly, surface finishes, such as anodization, can enhance emissivity and improve radiative cooling performance, especially if the heat sink operates in conjunction with a radiative cooling system. Considering all the factors mentioned above, the heat sink parameters were carefully selected, as presented in Table 3.

**Table 3:** Proprieties of the Air and Parameters of the Heat Sink

Parameter	Symbol	Value
The base area of the heat sink (mm^2^)	*A*_*b*_	1600
Density of air (kg m^–3^)	ρ	1.293
The inlet temperature of air (K)	*T*_*in*_	293
Length of a fin (mm)	*L*_*f*_	40
Heat capacity of air (kJ kg^–1^ K^–1^)	*C*_*p*_	1.007
Width of a channel (mm)	*w*_*ch*_	1.895
Heat transfer coefficient of air (W m^–2^ K^–1^)	*h*	20
Thickness of a fin (mm)	*t*	0.1
Height of a fin (mm)	*L*	20
Air velocity (m s^–1^)	*u*	1
Number of channels	*M*	20
Thermal conductivity of aluminum (W m^–1^ K^–1^)	*k*_*b*_	238
The thickness of the base of the heat sink (mm)	*L*_*b*_	0.5
Dynamic viscosity of air (kg m^–1^ s^–1^)	μ	1.846 × 10^–5^
Width of heat sink (mm)	*w*_*hs*_	40

In this study, real-time meteorological data from ASHRAE 2021 within COMSOL Multi-Physics is used to analyze the performance of the proposed system. To assess the model’s performance in different regions around the world, three cities with varying levels of solar insolation are chosen: Helsinki in Finland, Istanbul in Turkey, and Cairo in Egypt. The cities can be classified into three categories according to the sunshine intensity, namely mild, moderate, and warm. The ambient parameters are fetched from the meteorological data as follows:
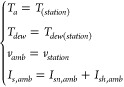
35where *I*_*sn,amb*_ is the direct solar irradiance and *I*_*sh,amb*_ is the diffuse solar irradiance.

For this study, the radiative cooler is assumed to have a solar reflectivity of approximately 90.7% in the visible spectrum and an emissivity of 90.11% in the sky window.^48^

## 3 Methodology

The geometry of the system is created with the material properties and physics nodes defined. Heat transfer node in solid and fluid is used to model the PCM, while the TE effects are modeled by the heat transfer in solid, electric current, and electric circuit nodes. Likewise, the heat transfer in solid and turbulent flow nodes modeled the heat sink, while the surface-to-surface radiation node solved the radiative cooling effect. The finite element numerical method is employed to analyze the proposed system in COMSOL Multi-Physics.

### 3.1 Boundary and Initial Conditions

- Presence of thermal paste at the interfaces between components.
- The side walls of the PCM box are assumed to have adiabatic conditions.
- The simulation begins with zero electric potential.
- Actual weather conditions are used throughout.
- Presence of radiation and convection losses.
- No slip at the wall and static pressure condition is used.
- Fully developed laminar flow and incompressible Newtonian fluid is assumed.

### 3.2 Grid-Independent Test

A grid-independent test is conducted to confirm the accuracy and consistency of the numerical simulations and to determine the appropriate mesh size that strikes a harmonious balance between accuracy and computational efficiency. The mesh size is determined by dividing the total volume of the model by the number of elements. For example, the total volume of the model is 69,560 mm^3^ when divided by the number of elements 103,019, the mesh size of 0.675 mm^3^ is obtained. The open circuit voltage of three models, namely PCM-TE-RC (radiative cooling with a windshield), PCM-TE-RC-conv (radiative cooling with free air convection on the cold side), and PCM-TE-RC-HS (radiative cooling coated on the heat sink), is determined while varying the mesh size, as shown in Table 4. A stationary simulation was conducted with a constant solar irradiance of 1000 Wm^2–^. It has been determined that the accuracy and computational time increase with a decrease in the mesh size. It can be concluded that the variation in mesh size has no significant effect on the results; therefore, the mesh size with 103,019 elements is chosen and used consistently.

**Table 4:** Grid-Independent Test Results Display the Open-Circuit Voltage of the Systems

	Number of elements			
System	174687	103019	51004	24278
PCM-TE-RC	38.5 mV	38.3 mV	38.1 mV	38.0 mV
PCM-TE-RC-conv	60.2 mV	60.1 mV	59.9 mV	59.9 mV
PCM-TE-RC-HS	1.32 V	1.32 V	1.32 V	1.32 V

## 4 Experimental Section

### 4.1 Synthesis of the Radiative Cooling Material

Graphite, TiO_2_, K_2_SiO_3_, BaSO_4_, CaCO_3_, and SiO_2_ (Sigma-Aldrich) were used to synthesize a two-layer radiative cooling material. The bottom layer, composed of graphite and K_2_SiO_3_, ensures high emissivity, while the top layer, consisting of TiO_2_, CaCO_3_, SiO_2_, K_2_SiO_3_, and BaSO_4_, is designed for high solar reflectivity. TiO_2_ provides the necessary reflectivity for radiative cooling, though it still absorbs some UV rays. To reduce UV absorption, CaCO_3_, SiO_2_, and BaSO_4_ are incorporated, and K_2_SiO_3_ serves as the binder. During the day, K_2_SiO_3_ loses moisture, tightening the particles in the top layer and enhancing solar reflectivity. At night, it absorbs moisture, loosening the particles and facilitating thermal transfer from the bottom layer to outer space. The bottom layer is composed of 1 g of graphite and 4 mL of K_2_SiO_3_, while the top layer is composed of 5 mL of K_2_SiO_3_, 0.04 g of SiO_2_, 1 g of TiO_2_, 0.16 g of CaCO_3_, and 0.8 g of BaSO_4_.

**Figure 2:**
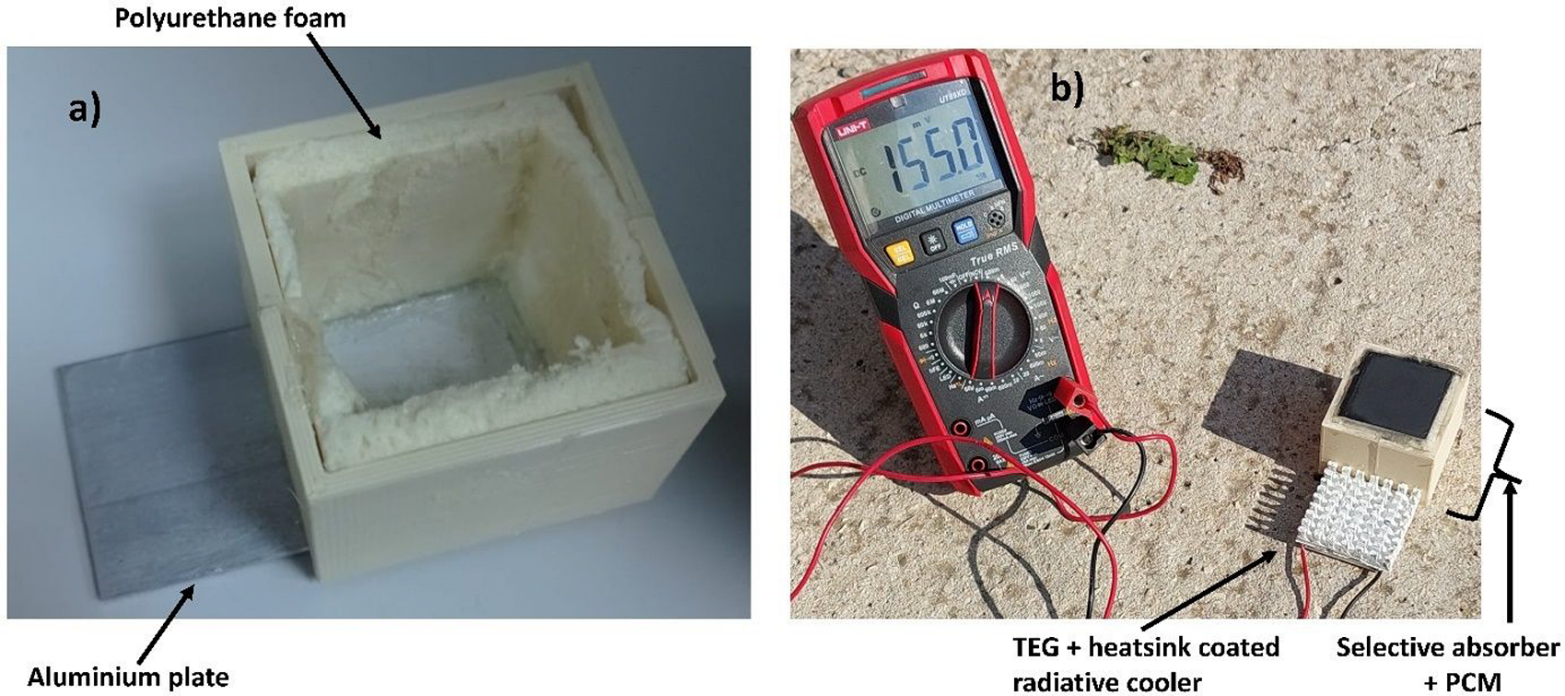
Experimental setup (a) during fabrication, (b) during measurement.

The synthesized RC material was coated on a small rectangular-plate heat sink, which was positioned on the cold side of a thermoelectric generator (TEC-12715). Figure 2a presents the fabrication process before placing the aluminum PCM box, and Figure 2b shows the completed experimental setup. The PCM box contains paraffin (CAS No: 8002–74–2) and a selective absorber, graphene (CAS No: 7782–42–5). The experiment was conducted on 24th September 2024, in Istanbul (41.0082° N, 28.9784° E).

## 5 Results and Discussion

### 5.1 Experimental Analysis

**Figure 3:**
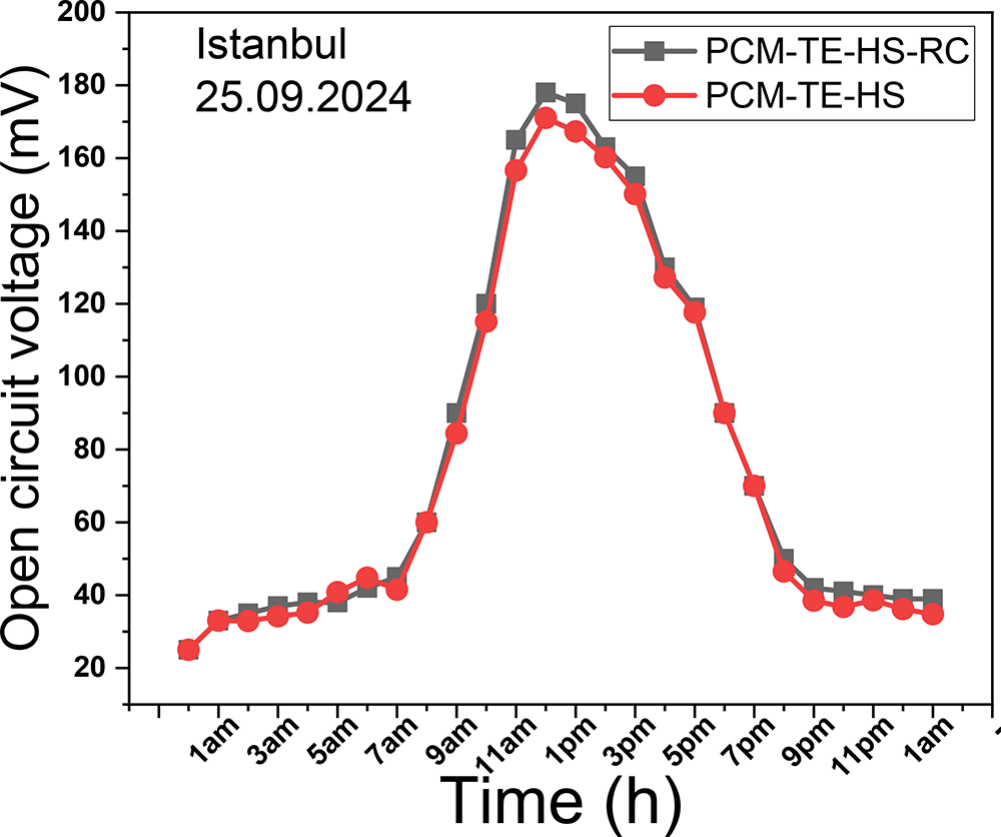
Open circuit voltage measurement.

Open circuit voltage measurements were performed on the PCM-TE-HS-RC and PCM-TE-HS (no radiative cooling) systems, as shown in Figure 3, with data sampled at 1-h intervals. The results show that the output voltages increased from around 20 mV in the morning to around 180 mV at noon, then decreased and stabilized at approximately 40 mV at nighttime. During the day, the temperature gradient reaches a maximum value of 7 K, while at night, it remains within the range of 2 to 3 K. The temperature gradient across the two sides of the TEG in the morning could be attributed to the heat sink alone or in combination with the radiative cooler. Throughout the day, the PCM played a crucial role in solar absorption, enhancing energy input to the TEG and thus improving performance. The PCM continued releasing its stored energy until it returned to a solid state at night, during which the output voltages remained stable at around 40 mV. A comparison of the two systems shows that the performance of the PCM-TE-HS-RC system is slightly better than that of the PCM-TE-HS system, demonstrating enhanced cooling due to the radiative cooler’s presence.

### 5.2 Theoretical Analysis

#### 5.2.1 Performance of the PCM-TE with Different Cooling Systems

The theoretical analysis is conducted to determine the effect of different parameters on the performance of the proposed system. Passive cooling is typically employed on the cold side to ensure a TEG’s long-term, maintenance-free operation in remote outdoor settings. In this context, an analysis of the PCM-TE system’s performance with different passive cooling methods: radiative cooling (PCM-TE-RC), radiative cooling with free air convection (PCM-TE-RC-Conv), and a heat sink that is air-cooled and coated with radiative cooling (PCM-TE-RC-HS) was conducted. The performance of each system using weather data from Istanbul (specifically, the data from the first day of each month in 2021, at noon) was evaluated to gain insights into their performance under different seasonal conditions.

**Figure 4:**
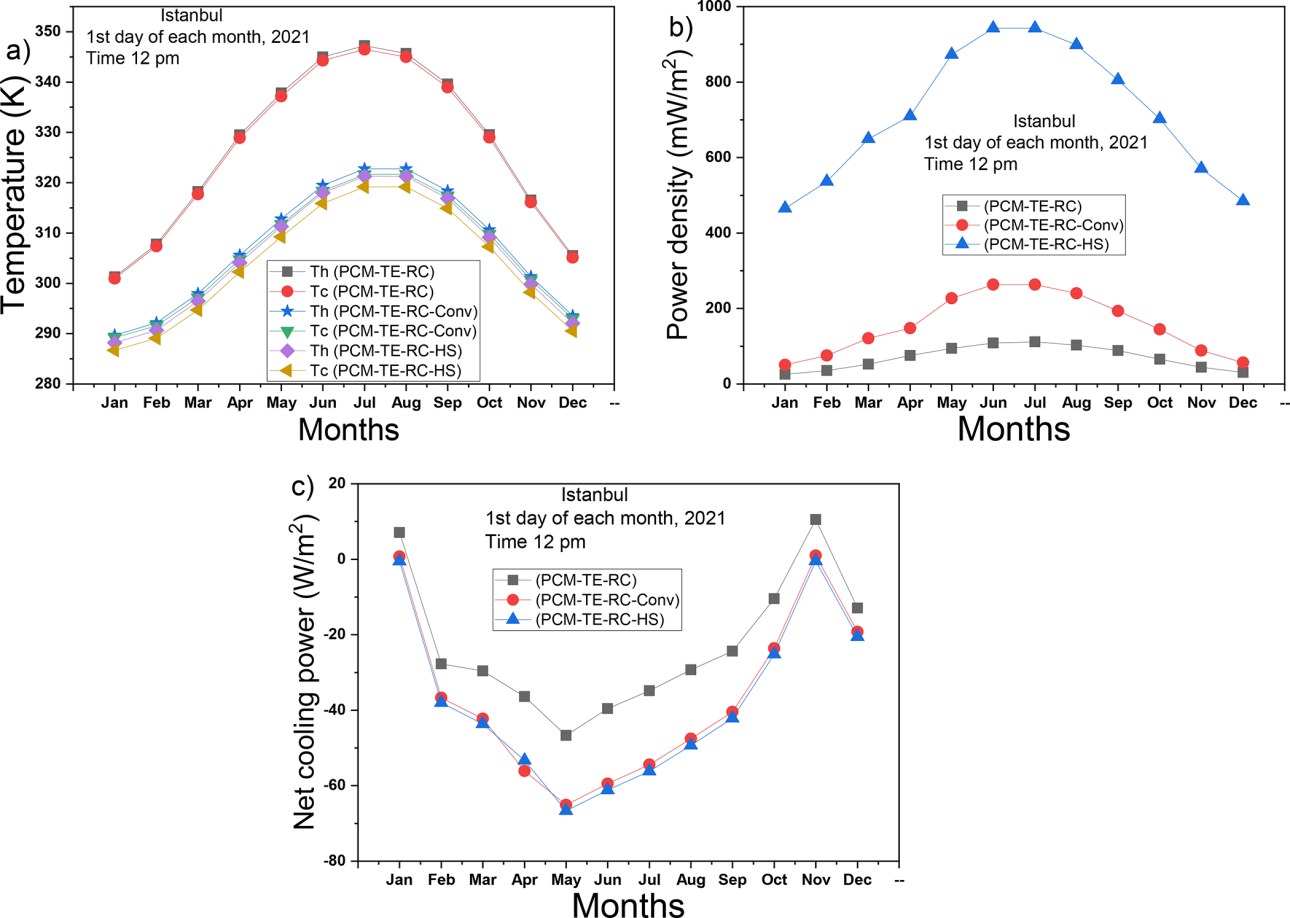
Performance comparison of the three systems: (a) temperatures of hot and cold sides, (b) power density of TEG, (c) net radiative cooling power of RC.

Figure 4a presents the variations in temperatures of the two surfaces of the TEG over the year. The temperatures reach their peak values during the summer months due to higher solar radiation and ambient temperature. The PCM-TE-RC system exhibits the lowest cooling performance and is associated with the highest temperatures. In contrast, the PCM-TE-RC-HS system displays the lowest temperatures and the most significant temperature gradient, thanks to its hybrid cooling approach. In Figure 4b, the power densities follow a normal distribution curve, with peaks occurring during the summer months. The PCM-TE-RC-HS, benefiting from enhanced cooling performance, outperforms the PCM-TE-RC system by more than 10-fold and the PCM-TE-RC-Conv system by approximately 4-fold. This result underscores the beneficial impact of both radiative and convective cooling on the TEG’s output performance.

Figure 4c presents the net cooling power of radiative cooling in all three systems. The PCM-TE-RC system exhibits the highest net radiative cooling, while the PCM-TE-RC-HS system has the lowest. This difference arises because the presence of convection has a detrimental effect on the radiative cooler’s performance. In the PCM-TE-RC system, convection cooling is assumed to be nonexistent, as the windshield is incorporated on the TEG’s cold side. In the PCM-TE-RC-Conv system, the windshield is removed, allowing natural air to flow across the TEG’s cold side. For the PCM-TE-RC-HS system, a heat sink is coated with radiative cooling and integrated into the TEG’s cold side. It can be seen that the net radiative cooling of the PCM-TE-RC and PCM-TE-RC-Conv systems achieved positive values in January and November. In summary, while convection cooling reduces the net radiative cooling power of the radiative cooler, it enhances the TEG’s output performance. Consequently, the PCM-TE-RC-HS system proves superior to the other systems and is chosen for subsequent analyses.

#### 5.2.2 Impact of Melting Temperature on the Performance of PCM-TE-RC-HS System

**Figure 5:**
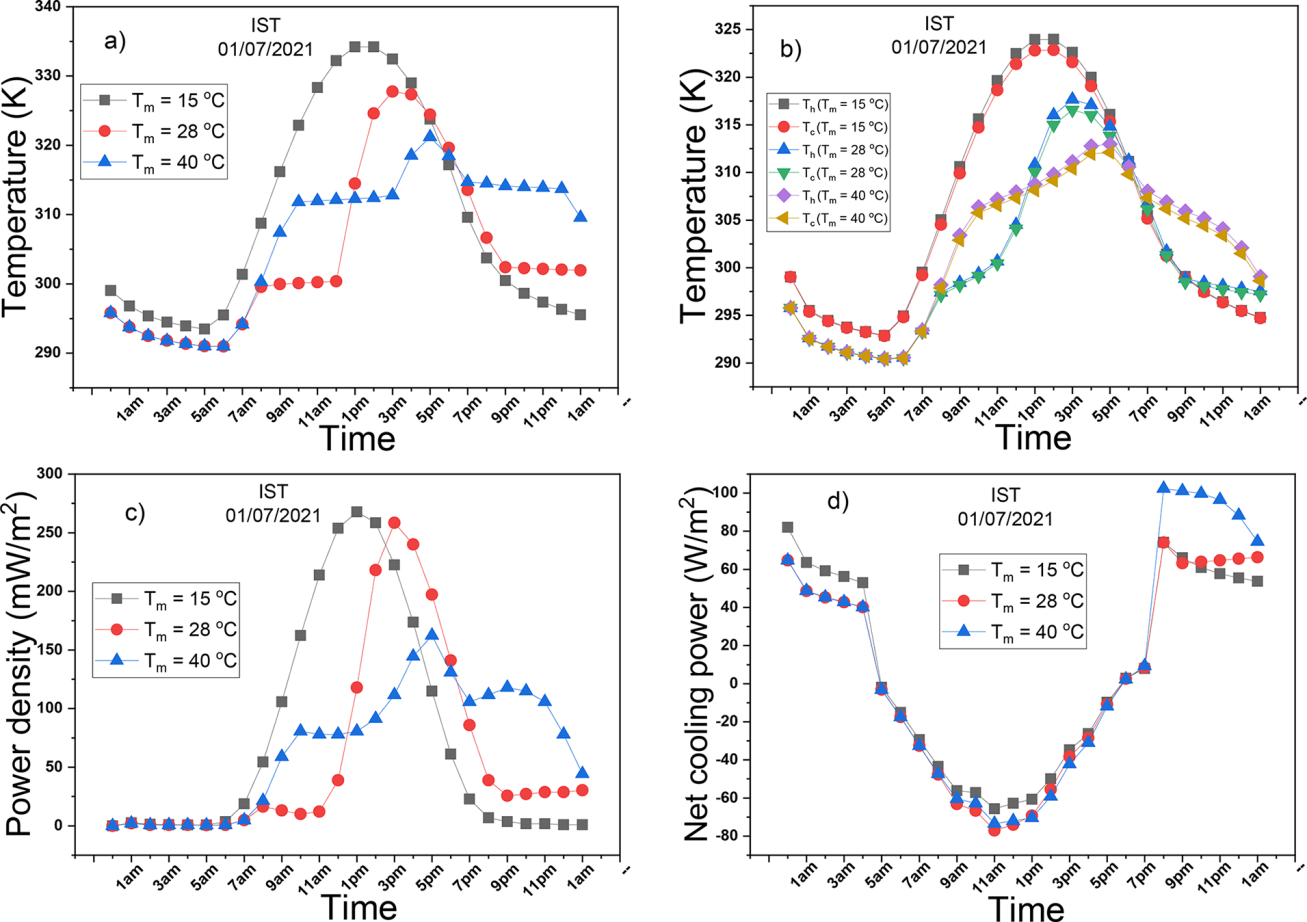
Effect of variation of the melting temperature of PCM on (a) temperature of PCM, (b) hot and cold sides’ temperatures, (c) power density of TE, (d) net radiative cooling power of RC.

The objective of this analysis is to investigate the effect of *T*_*m*_ on the performance of the PCM-TE-RC-HS system. An analysis with three distinct *T*_*m*_ values: 15 °C, 28 °C, and 40 °C was conducted. These *T*_*m*_ values were intentionally selected to assess how the PCM performs when its melting temperature is significantly below, within the ambient temperature range, and significantly above it. The city of Istanbul is selected as a case study where its ambient temperature on first July 2021, lies between 20 and 25 °C, which means that the *T*_*m*_ values of 15 and 40 °C fall below and above the ambient temperature range. The temperature of the PCM is assumed to be at the center of the PCM container. In Figure 5a, the variations in the PCM’s temperature over the simulation period are presented. In the first few hours, the PCM temperatures in all cases slightly decrease because the PCM is not yet functional due to the absence of solar radiation. As the sun rises, the PCM with *T*_*m*_ = 15 °C experiences a rapid temperature increase, reaching its peak before decreasing. This suggests that the PCM with *T*_*m*_ = 15 °C has already undergone the phase transition due to the ambient temperature being above the melting temperature. In contrast, the PCM with *T*_*m*_ = 28 and 40 °C undergo phase transition when their temperatures reach 301 and 313 K, respectively. This phase transition occurs at a constant temperature, during which a substantial amount of energy is absorbed and stored by the PCM. This explains why the PCM with *T*_*m*_ = 28 and 40 °C maintain constant temperatures during phase transitions.

Figure 5b presents the variations in temperatures of the hot and cold sides over the simulation period. These plots resemble the PCM’s temperature plot, as the PCM has a significant influence on the temperatures of the two sides of the TEG. Temperature gradients are observed during the daytime and nighttime when the phase transition occurs. These temperature gradients are due to the presence of thermal energy on the hot side and heat sink-coated radiative cooler on the cold side of the TEG. The PCM with *T*_*m*_ = 15 °C, not undergoing a phase transition, displays a minimal temperature gradient. Figure 5c presents the variations in power densities over the simulation period for all three cases. In the initial 6 h of the simulation, power densities are low due to low-temperature gradients. As the sun rises, the power density of the PCM with *T*_*m*_ = 15 °C rapidly increases, reaches a peak, and then decreases abruptly. In contrast, the power densities of the PCM with *T*_*m*_ = 28 and 40 °C gradually rise, remain constant during the phase transformation periods (solid to liquid), reach their maximum values, and then reduce, remaining constant during the phase transition periods (liquid to solid). The highest daytime power densities of the TEG operating with the PCM with *T*_*m*_ = 15 °C, 28 °C, and 40 °C are 267 mW/m^2^, 258 mW/m^2^, and 162 mW/m^2^, respectively. Similarly, the highest nighttime power densities of the TEG operating with the PCM with *T*_*m*_ = 15 °C, 28 °C, and 40 °C are 1 mW/m^2^, 30 mW/m^2^, and 100 mW/m^2^, respectively. The nighttime power densities are due to the energy released by the PCM. When the melting temperature is high, the hot side temperature of the TEG is also high, resulting in a significantly high power density. Although the diurnal power density of the TEG operating with PCM with *T*_*m*_ = 15 °C is slightly higher than that of the PCM with *T*_*m*_ = 28 °C, its lack of nighttime output makes it less appealing. Comparing the nocturnal power densities of the TEG operating with PCM with *T*_*m*_ = 28 and 40 °C, the former remains stable, while the latter decreases as the PCM loses thermal energy. The TEG operating with PCM with *T*_*m*_ = 28 °C has the overall highest average performance, and is, therefore, the most suitable melting temperature of the PCM operating in Istanbul in the summer. This melting temperature is used in the subsequent analyses.

Figure 5d displays the variations in net radiative cooling power throughout the simulation period. Since there is no solar energy at night, the net radiative cooling powers are positive. As the sun rises, the net radiative cooling powers fall to negative values due to the inevitable solar absorption by the radiative cooler. Initially, the PCM with *T*_*m*_ = 15 °C has the highest net radiative cooling power, while in the final 6 h of the simulation, the PCM with *T*_*m*_ = 40 °C has the highest net radiative cooling power. In summary, the *T*_*m*_ exerts a significant influence on the PCM-TE-RC-HS system’s operation. Therefore, careful consideration must be given when selecting a PCM with the appropriate melting temperature.

#### 5.2.3 Impact of Varying Radiative Cooler Reflectivity on PCM-TE-RC-HS System Performance

In this study, a benchmark radiative cooler with approximately 90% reflectivity between 0.3–2.5 μm is employed. Since achieving significant radiative cooling during the daytime can be challenging, the effect of altering the diurnal reflectivity of the radiative cooler on the PCM-TE-RC-HS system’s performance is investigated. The cases are delineated as follows: (0–0.5 μm Emiss 0.1) signifies that the radiative cooler has a 90% reflectivity in the 0–0.5 μm wavelength range and high absorptivity in the 0.51–2.5 μm wavelength range, with nocturnal optical properties remained unaltered. Restricting high reflectivity to the 0–0.5 μm wavelength range implies that solar energy will be absorbed in other solar spectrum regions. Similarly, (0–1.5 μm Emiss 0.1) denotes that the radiative cooler has a 90% reflectivity in the 0–1.5 μm wavelength range and high absorptivity in the 1.51–2.5 μm wavelength range, with nocturnal optical properties unchanged from the benchmark radiative cooler.

**Figure 6:**
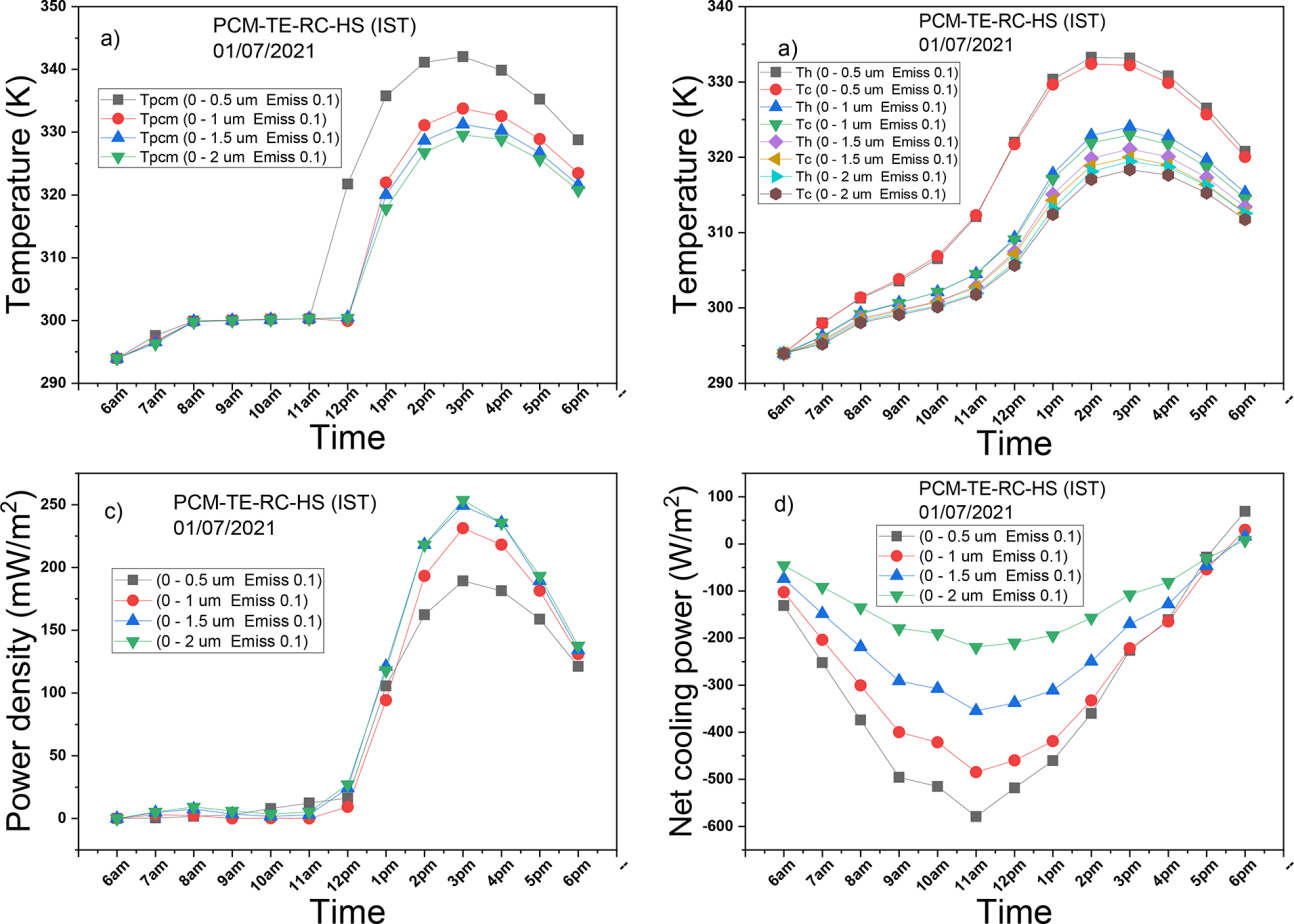
Effect of variation of the emissivity of the RC on (a) temperature of PCM, (b) hot and cold sides’ temperatures, (c) power density of TE, (d) net radiative cooling power of RC.

Figure 6a illustrates the variation of the PCM’s temperature over the simulation period, which exclusively covers the daytime since the nocturnal properties of the radiative cooler remain the same as the original radiative cooler. From the early morning hours until approximately 11 am, the PCM maintains temperature control in all cases. Subsequently, the PCM’s temperature experiences a sharp rise in the case with high solar absorptivity of the radiative cooler (0–0.5 μm Emiss 0.1). Around noon, the temperatures of the PCM in the other three cases also rise, reaching their peak values before gradually decreasing. The temperature of the PCM decreases as solar absorption by the radiative cooler decreases; in this scenario, the radiative cooler with (0–2 μm Emiss 0.1) results in the lowest temperature. Figure 6b illustrates the variations in the hot and cold sides temperatures over the simulation period, influenced by the reasons explained earlier. The system with the radiative cooler (0–0.5 μm Emiss 0.1) exhibits the highest temperatures on both the hot and cold sides. These temperatures have similar behaviors as that of the PCM’s, this is because the PCM serves as the TEG’s heat source; thus, it has great influence on the temperatures of the TEG. A closer look reveals negligible temperature gradients from the simulation’s start until around noon. Subsequently, temperature gradients emerge in the systems, with the system possessing the highest solar reflectivity (0–2 μm Emiss 0.1) exhibiting the most significant temperature gradient.

Following the temperature gradient plot, the power density plot is derived, as shown in Figure 6c. In the initial 6 h of the simulation, the power density in each case hovers around zero. However, in the last 6 h, the power density in each case suddenly surges to peak values before gradually decreasing. The maximum power densities for systems with high solar reflectivity in the wavelength range of 0–0.5 μm, 0–1 μm, 0–1.5 μm, and 0–2 μm are 189 mW/m^2^, 231 mW/m^2^, 249 mW/m^2^, and 254 mW/m^2^, respectively. Figure 6d displays the variation in net radiative cooling power throughout the simulation period. As anticipated, the system with the highest solar absorptivity (0–0.5 μm Emiss 0.1) exhibits the least radiative cooling performance, while the system with the highest solar reflectivity (0–2 μm Emiss 0.1) displays the highest net radiative cooling. When the two cases are compared, the net diurnal radiative cooling of the latter is enhanced by 164%. Due to the combination of solar absorption and convection cooling, which both negatively affect the radiative cooling performance, the net radiative cooling power for each case is negative except during the last hour of the simulation.

#### 5.2.4 Seasonal Performance of the PCM-TE-RC-HS

**Figure 7:**
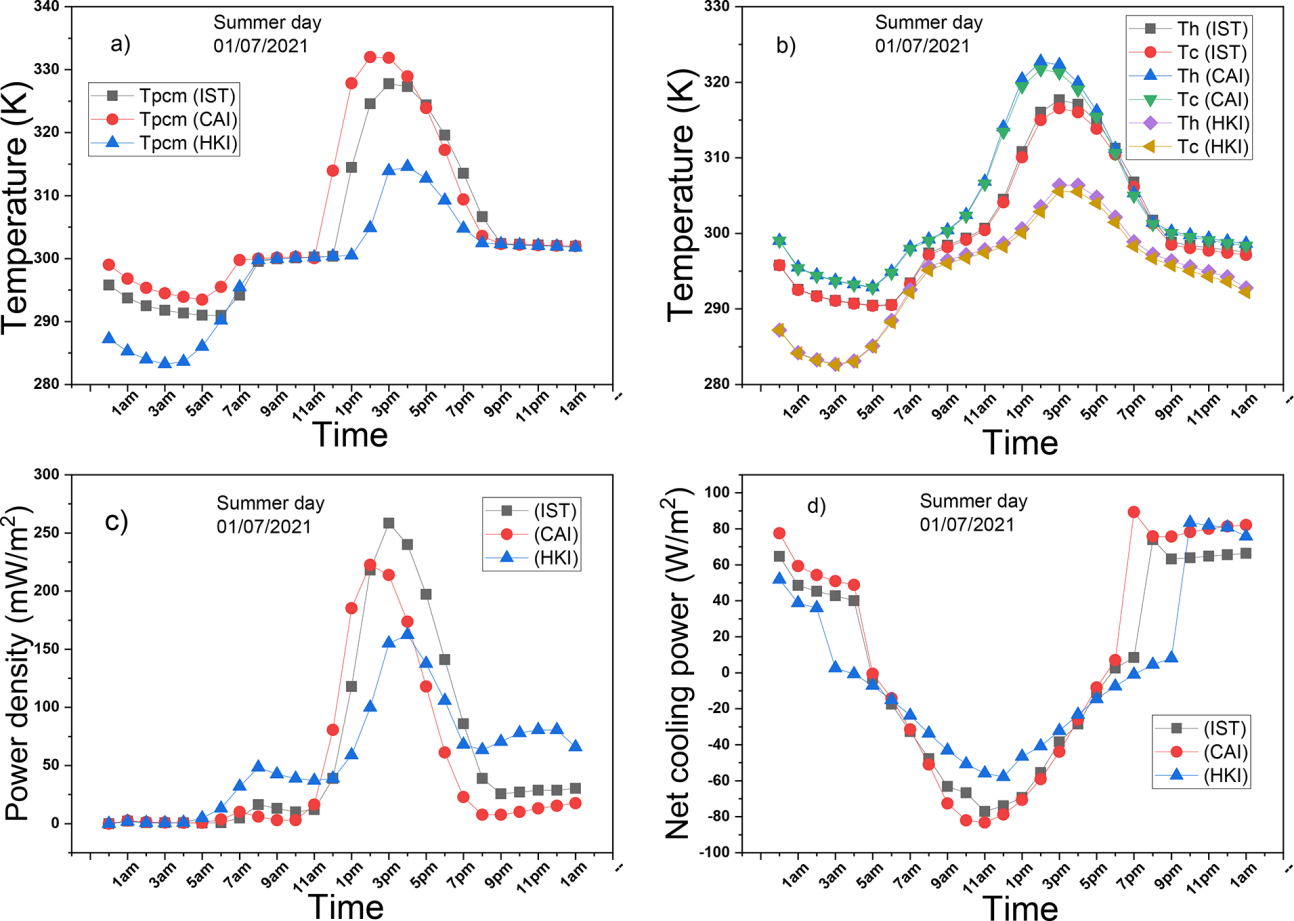
Performance of the PCM-TE-RC-HS on a summer day: (a) temperature of PCM, (b) hot and cold sides’ temperatures, (c) power density of TE, (d) net radiative cooling power of RC.

The summer and winter performances of the proposed system operating in Istanbul (Turkey), Helsinki (Finland), and Cairo (Egypt) are determined. This is done to evaluate the performance of the proposed system during different periods of the year. The system’s performance simulated on a summer day in the three cities is depicted in Figure 7 where the sample of the weather data is presented in the Supporting Information (Figure S1).

Figures 7a and 7b illustrate the PCM’s temperature as well as the temperatures of the hot and cold sides for the PCM-TE-RC-HS system operating in Istanbul (IST), Cairo (CAI), and Helsinki (HKI). Over the first few hours, all temperatures match the ambient temperature of their respective cities since the PCM is not yet operational. As the sun rises, PCMs’ temperatures gradually rise and stabilize at 301 K. During this period, the PCMs consistently absorb and store thermal energy. With further increases in solar radiation, PCMs’ temperatures continue to rise, reaching their respective peaks at different times. Subsequently, temperatures decrease and stabilize at 302 K, which falls within the phase transition range. At this temperature, the PCMs release thermal energy. The PCM in CAI operates at a higher temperature than the other two cities, owing to the higher solar radiation and ambient temperature in CAI. Figure 7b follows a similar pattern to Figure 7a, in addition, temperature gradients peak during daytime hours because the TEG receives maximum energy, and the cold side benefits from radiative cooling via a coated heat sink.

Figure 7c portrays the variation of power density over the simulation period. Upon comparing the power densities, it is evident that the systems operating in IST, CAI, and HKI generate power densities of 258 mW/m^2^, 222 mW/m^2^, and 162 mW/m^2^, respectively. The system operating in IST achieves the highest average power density due to favorable weather conditions. During the early morning and nighttime hours, the power density of the system operating in HKI surpasses that of the other scenarios, as HKI experiences longer sunshine hours, receiving solar radiation for an extended period. A noteworthy observation is that all systems generate power density during nighttime in the last hours of the simulation, thanks to the energy released by the PCM. The presence of the PCM and a heat sink coated with radiative cooling enables the TEG to operate continuously for 24 h. Figure 7d illustrates the variation of net radiative cooling power over the simulation period. The net radiative cooling power is negative during the daytime and positive at night, as solar energy is absent during the nighttime hours. The system in CAI exhibits the most effective radiative cooling performance at night but performs less effectively during the daytime due to challenging weather conditions.

**Figure 8:**
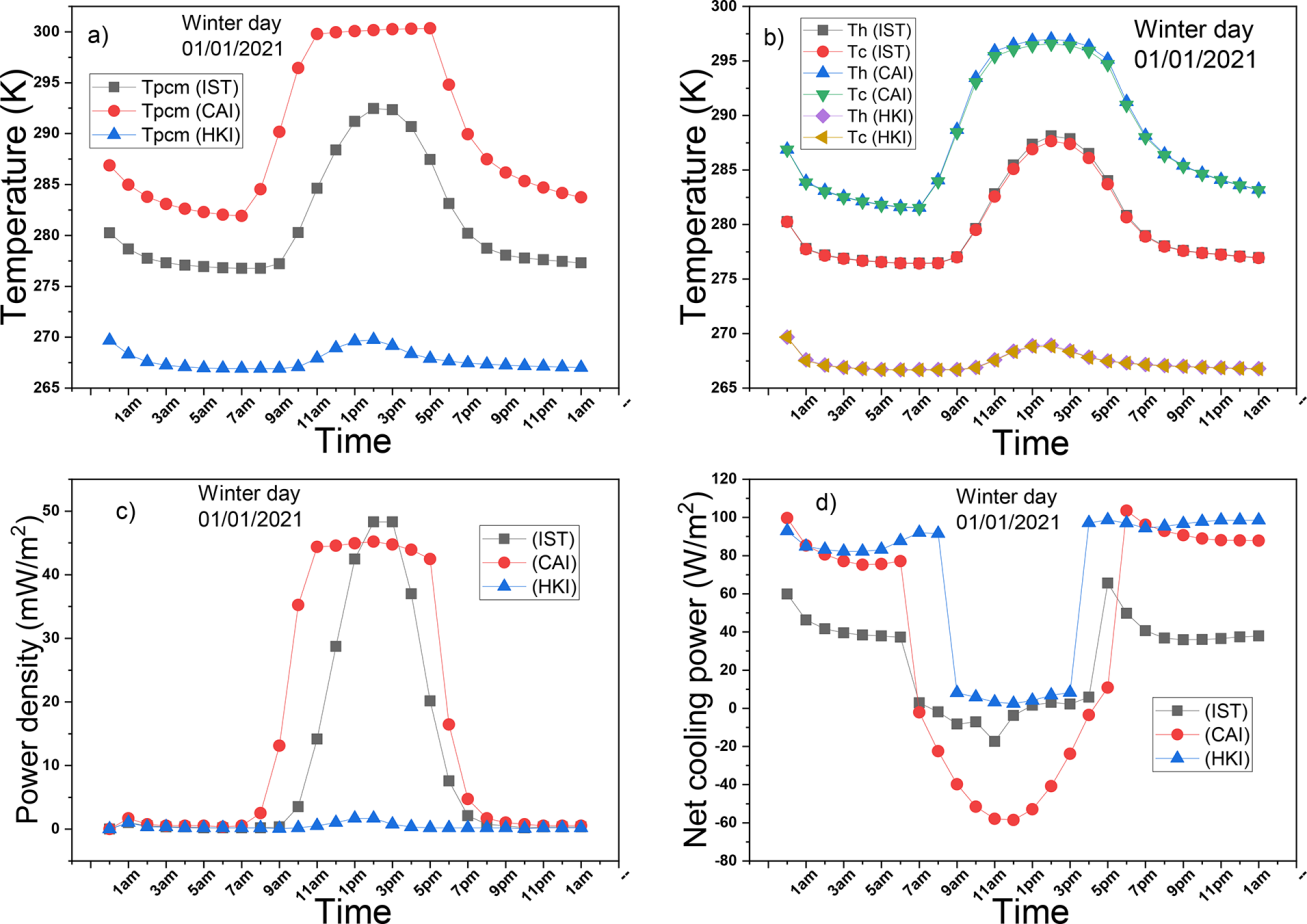
Performance of the PCM-TE-RC-HS on a winter day: (a) temperature of PCM, (b) hot and cold sides’ temperatures, (c) power density of TE, (d) net cooling power of RC.

Figure 8a illustrates the changes in PCM temperature over the simulation period on a winter day in the three cities. The variations in PCM temperatures are primarily attributed to varying levels of solar insolation in these locations. Remarkably, there are substantial discrepancies in PCM temperatures among the cities. In HKI, the PCM temperature remains consistently below 270 K throughout the simulation. In IST, it remains below the melting temperature, and in CAI, it barely reaches the melting temperature. It is important to note that these PCM temperature variations have a significant impact on the hot and cold sides of the TEG, as depicted in Figure 8b. In all three cities, the temperature gradients across the TEG sides are notably lower than those observed during summer days. Consequently, the power densities, as shown in Figure 8c, are correspondingly lower compared to those in the summer. The system operating in HKI generates very low power density, while the system operating in IST reaches a peak power density of 48 mW/m^2^ around 1 pm. In contrast, the highest power density generated by the system operating in CAI is 45 mW/m^2^, which remains almost constant for 7 h during the day. The peak power density of the TEG operating in IST is superior to that of the TEG operating in CAI due to the higher temperature gradient across its two surfaces between 1 to 2 pm. The power densities generated at night are solely a result of radiative cooling, as the PCM temperatures in all the cities remain below the melting temperature during the night. Figure 8d depicts the changes in the net radiative cooling power over the simulation period. Due to the low solar insolation in IST and HKI, high net radiative cooling powers are observed, with the system operating in HKI reaching as high as 98 W/m^2^ and maintaining non-negative values throughout. For the system operating in IST, the net radiative cooling reaches up to 60 W/m^2^ with occasional negative values, including a peak of −17 W/m^2^. In contrast, the system operating in CAI experiences the least favorable diurnal net radiative cooling due to its high solar insolation.

#### 5.2.5 Evaluation of the Practicality of Implementing the Proposed PCM-TE-HS-RC System

A simulation was performed in LTSpice to assess the feasibility of using the proposed PCM-TE-HS-RC system to power a DC-DC converter (LTC3108–1), and the schematic is provided in the Supporting Information (Figure S2). The DC-DC converter requires a minimum input of 20 mV to initiate operation. The simulation assumes that the PCM-TE-HS-RC system can generate at least 20 mV output voltage. To enhance system reliability, the setup is scaled up by thermally connecting four TEGs in parallel, with pairs of TEGs electrically connected in series, and their outputs electrically connected in parallel. The series arrangement increases the output voltage, while the parallel connection boosts the electric current and reduces the source impedance. The system is designed to be deployed remotely to power a digital temperature sensor (STT75), which has a load requirement of 75 μA during bursts and almost zero load between bursts. A capacitor (C_out_) connected in parallel with the load will supply or absorb current to maintain the voltage across it. When the load current suddenly increases (during the pulse), the capacitor initially provides the extra current needed by the load. Conversely, when the load current suddenly decreases, the capacitor absorbs the excess current. The value of this capacitor can be determined as follows:
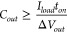
36where *I*_*load*_ is the total load current during the burst, *t*_*on*_ is the duration of the burst, *ΔV*_*out*_ is the maximum allowable voltage droop.

The storage capacitor that will drive the sensor when there is no input from the source can be sized as follows:
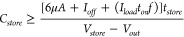
37where *I*_*off*_ is the load on the *V*_*out*_ during the sleep mode, 6 μA is the quiescent current of the LTC3108–1, *V*_*store*_ is the storage voltage, *f* is the frequency of the burst, *t*_*store*_ is the storage time, which is assumed to be 24 h in this study.

The time needed to charge the *C*_*out*_ and *C*_*store*_ at the initial start of the system can be respectively given as
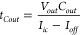
38
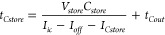
39where *I*_*cstore*_ is the charge current required to charge the *C*_*store*_ and *I*_*ic*_ is the charge current supplied by the LTC3108–1, which is given by
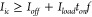
40

**Figure 9:**
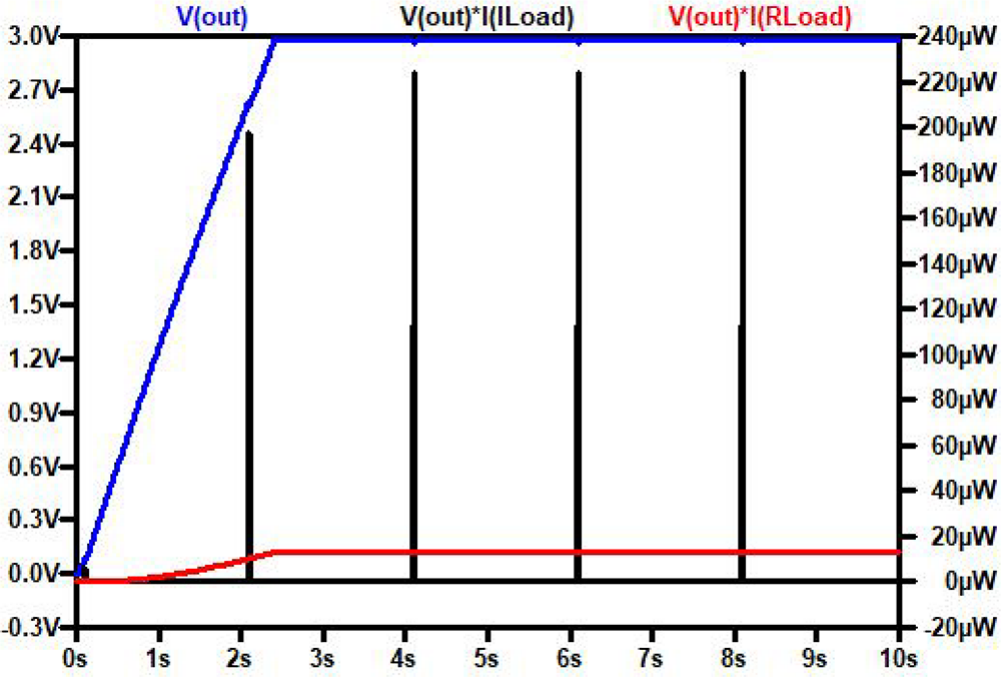
Transient simulation of the LTC3108–1 powered by the PCM-TE-HS-RC system.

Figure 9 shows the transient plot of the output voltage, output power during the sleep mode (*V*_out_*I*_Rload_), and output power during the burst (*V*_out_*I*_Iload_). In reality, the burst occurs every 30 min, but for the simulation, it is set to occur every 2 s. It can be seen that *C*_*out*_ achieves voltage regulation of 3 V in just 2.3 s. Before this, the power requirement of the temperature sensor is not met. However, once voltage regulation is achieved, *C*_*out*_ adjusts the electric current in the circuit to maintain a constant output voltage, accommodating both steady and transient loads. Although the source operates continuously for 24 h, a backup capacitor (C_store_) can supply power when there is no output from the TEGs.

## 6 Conclusions

A thorough examination of a system combining PCM with a TEG along with a heat sink coated with a radiative cooler is presented. The system is configured such that the cold side of the TEG benefits from both convection and radiative cooling. The investigation delves into the influence of key parameters on the operation and overall performance of the system, yielding several noteworthy findings, which can be summarized as follows:

- The performance of the TEG experiences a remarkable improvement through integration with a heat sink coated with radiative cooling technology. Particularly, a 10-fold enhancement in performance is achieved when the cold side of the TEG is coupled with a heat sink-coated radiative cooler compared to relying solely on radiative cooling.
- The melting temperature of the PCM performs a vital role in the nighttime operation of a TEG. For the TEG to operate effectively at night, it is imperative that the PCM can release energy.
- To achieve robust diurnal radiative cooling performance, a radiative cooler with high reflectivity across the entire solar spectrum is highly desirable. The study reveals that extending the wavelength range covered by the radiative cooler’s reflectivity from 0.5 to 2 μm enhances the net diurnal radiative cooling by 164%.
- On a summer day, the PCM-TE-RC-HS system exhibits the capability to generate substantial power densities, reaching maximum values of 258 mW/m^2^ in IST, 222 mW/m^2^ in CAI, and 162 mW/m^2^ in HKI. Moreover, the system demonstrates the capacity to produce reasonable power densities at night, underscoring its suitability for uninterrupted 24-h operation during the summer.
- The PCM-TE-HS-RC system is capable of powering sensors located remotely.

This compact system exhibits great potential for remote applications where continuous, low-energy, and extended operation is desired.

## Data Availability

Data will be made available on request.

## Supporting Information Available

Figures of real weather data for simulation and schematic of power management circuit (PDF)
