# Supplementary material for: Numerical and Experimental Analyses of a Phase Change Material-Thermoelectric System Integrated with a Heat Sink and Radiative Cooling
Source: ACS Appl Mater Interfaces. 2024 Dec 13;16(51):70701–15. doi: 10.1021/acsami.4c17331 (PMC11672478; doi:10.1021/acsami.4c17331)
Supplement: Supplementary file 1 — am4c17331_si_001.pdf [file am4c17331_si_001.pdf]

Supporting Information

Numerical and experimental analyses of a phase change material-thermoelectric system integrated with a heat sink and radiative cooling

Aminu Yusuf [a], Sedat Ballikaya [a*]

[a] Department of Engineering Sciences, Istanbul University-Cerrahpasa, Avcilar, Istanbul, 34320, Turkey

Correspondence: ballikaya@iuc.edu.tr

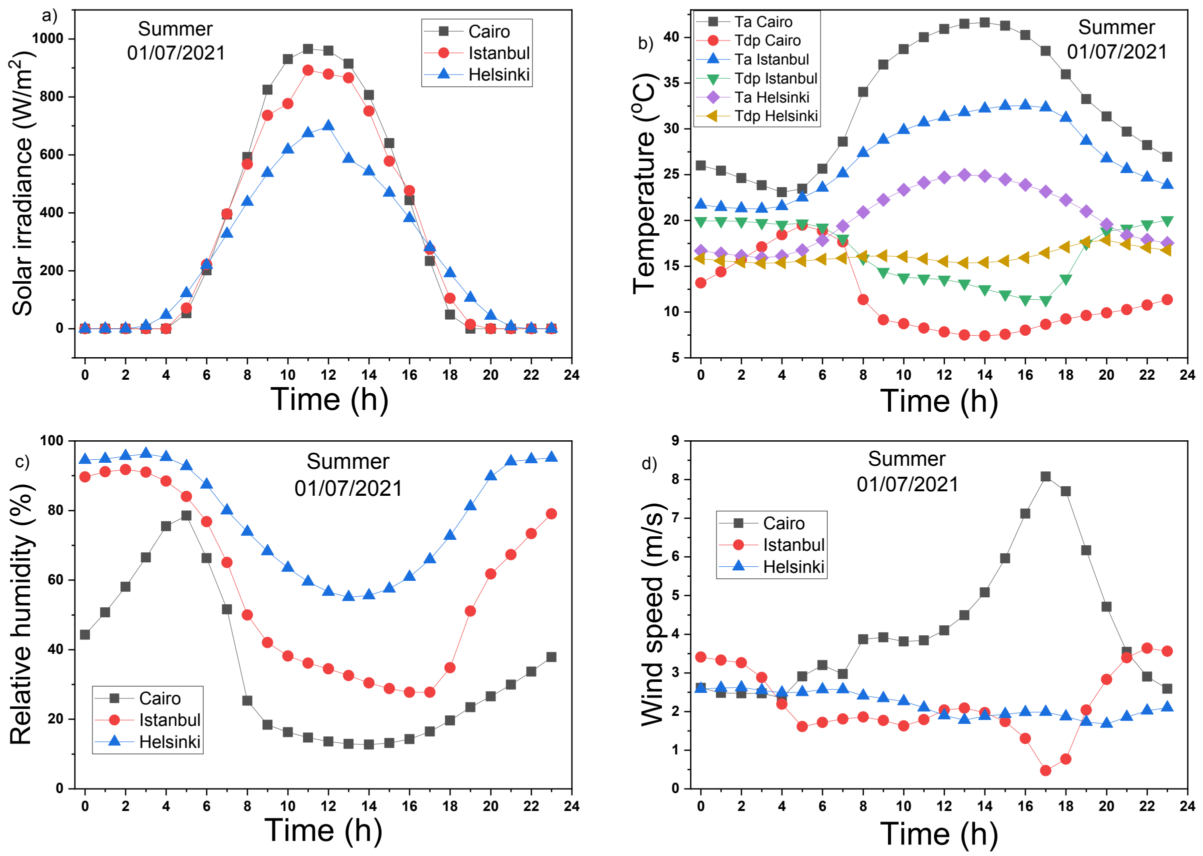

Fig. S1. Sample hourly weather data of the three cities used in the simulation (01/07/2021): a) Solar irradiance, b) Ambient and dew point temperatures, c) relative humidity, d) wind speed.

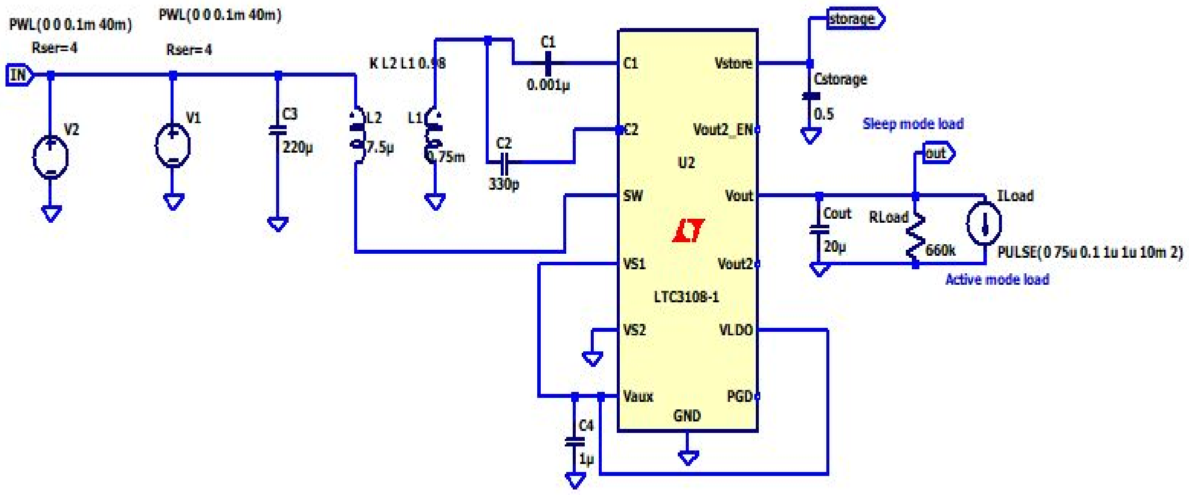

Fig. S2: Schematic of the power management circuit.
